# Comparative transcriptome analysis of hESC- and iPSC-derived lentoid bodies

**DOI:** 10.1038/s41598-019-54258-z

**Published:** 2019-12-06

**Authors:** Muhammad Ali, Firoz Kabir, Jason J. Thomson, Yinghong Ma, Caihong Qiu, Michael Delannoy, Shahid Y. Khan, S. Amer Riazuddin

**Affiliations:** 1 The Wilmer Eye Institute, Johns Hopkins University School of Medicine, Baltimore, MD 21287 USA; 2 Yale Stem Cell Center, Yale University School of Medicine, New Haven, CT 06520 USA; 3 Department of Cell Biology and Imaging Facility, Johns Hopkins University School of Medicine, Baltimore, MD 21205 USA

**Keywords:** Transcriptomics, Embryonic stem cells

## Abstract

The ocular lens serves as an excellent system to investigate the intricate details of development and differentiation. Generation of lentoid bodies or lens-like structures using pluripotent stem cells is important for understanding the processes critical for lens morphogenesis and the mechanism of cataractogenesis. We previously reported the generation of peripheral blood mononuclear cell (PBMC)-originated, induced pluripotent stem cells (iPSCs). Here, we report generation of lentoid bodies from human embryonic stem cells (hESCs) and (PBMC)-originated, iPSCs employing the “fried egg” method with brief modifications. The ultrastructure analysis of hESC- and iPSC-derived lentoid bodies identified closely packed lens epithelial- and differentiating fiber-like cells. In addition, we performed RNA sequencing (RNA-Seq) based transcriptome profiling of hESC- and iPSC-derived lentoid bodies at differentiation day 25. Next-generation RNA sequencing (RNA-Seq) of hESC- and iPSC-derived lentoid bodies detected expression (≥0.659 RPKM) of 13,975 and 14,003 genes, respectively. Comparative transcriptome analysis of hESC- and iPSC-derived lentoid bodies revealed 13,563 (>96%) genes common in both datasets. Among the genes common in both transcriptome datasets, 12,856 (~95%) exhibited a quantitatively similar expression profile. Next, we compared the mouse lens epithelial and fiber cell transcriptomes with hESC- and iPSC-derived lentoid bodies transcriptomes and identified > 96% overlap with lentoid body transcriptomes. In conclusion, we report first-time comparative transcriptome analysis of hESC- and iPSC-derived lentoid bodies at differentiation day 25.

## Introduction

The ocular lens offers an excellent system to explore the complex process of development and differentiation. The ocular lens originates from the head ectoderm, which thickens to generate the lens placode during embryonic development^1,2^. The lens placode internalizes the optic vesicle constituting the lens pit that separates from the ectoderm to develop lens vesicle. Subsequently, lens vesicle divides into two single-cell layers. The anterior layer forms the lens epithelium while cells of the posterior layer give rise to primary fiber cells^1,2^.

Differentiation of pluripotent stem cells to lentoid bodies or lens-like structures is important for understanding lens development and investigating the processes critical for lens morphogenesis. Previously, lentoid bodies have been successfully generated from human embryonic stem cells (hESCs) and induced pluripotent stem cells (iPSCs)^3,4^. Yang and colleagues differentiated hESCs into lens progenitor cells to form lentoid bodies directed by chemically defined conditions^3^. These lentoid bodies expressed lens-associated markers; however, they lack the light focusing ability^3,4^. Fu and colleagues developed lentoid bodies from human urinary epithelial cell-originated, iPSCs employing the “fried egg” method of differentiation^4^. These lentoid bodies expressed lens-associated markers and displayed transparent structure like a human lens^4^. Murphy and colleagues recently reported the generation of human micro-lenses by differentiating pluripotent stem cells into spheroidal masses of human lens epithelial-like cells^5^.

Pluripotent stem cell-derived lentoid bodies have tremendous potential to investigate the mechanism of human lens development and age-related cataractogenesis. Herein, we report RNA sequencing (RNA-Seq) based transcriptome profiling of lentoid bodies derived from hESCs and peripheral blood mononuclear cell (PBMC)-originated, iPSCs.

## Results

### Reprogramming to induced pluripotency

The PBMCs were isolated using 10 ml blood aliquot from a 67-year-old healthy male, with normal vision and reprogrammed with an integration-free Sendai virus gene delivery method as reported previously^6^. The PBMC-originated, iPSC colonies were selected and assessed for the expression of pluripotent markers by quantitative real-time polymerase chain reaction (qRT-PCR) and flow cytometry (Supplementary Fig. 1). The qRT-PCR analysis revealed high levels of pluripotent markers i.e. *NANOG, OCT4, SOX2* and *TRA-1-60* (Supplementary Fig. 1b). The flow cytometry analysis confirmed that PBMC-originated, iPSCs were positive for SSEA4 (94.56 ± 2.43%) and TRA-1-60 (89.36 ± 1.57%) (Supplementary Fig. 1c,d). Cryopreservation of PBMCs did not affect the reprogramming efficiency to induce pluripotency.

### Generation of lentoid bodies

**Figure 1:**
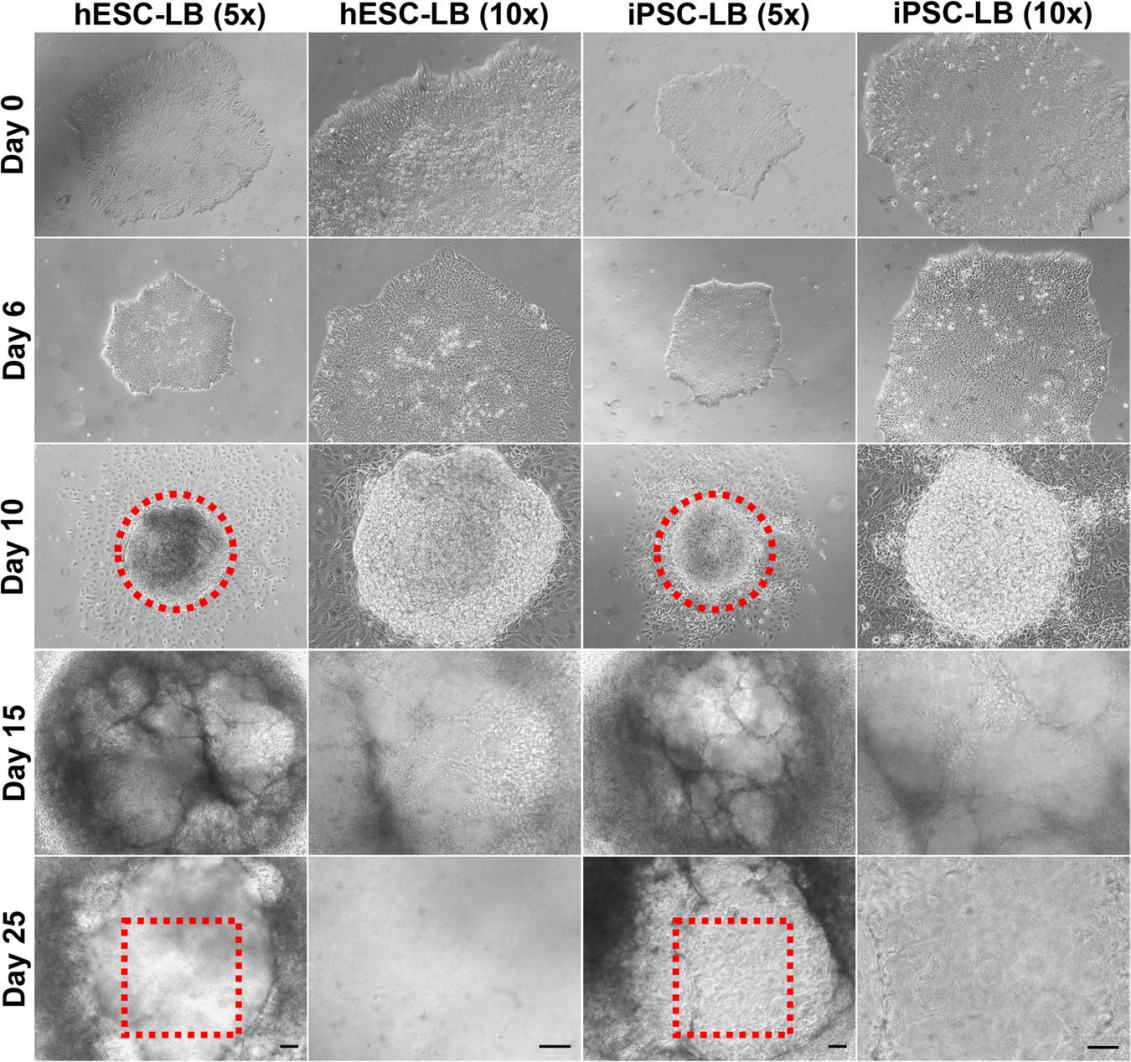
Generation of lentoid bodies (LB) from H9 human embryonic stem cells (hESCs) and peripheral blood mononuclear cell (PBMC)-originated, induced pluripotent stem cells (iPSCs). Phase-contrast microscopy at various magnifications and time points during LB differentiation at days 0, 6, 10, 15, and 25. The red dotted circles represent the “fried egg” morphology on day 10 while the red dotted squares indicate a lens-like transparent structure on day 25. The images are of 5x and 10x magnifications and the scale bars represent 100 µm.

H9 hESCs and PBMC-originated, iPSCs were subjected to a 25-day differentiation procedure to develop lentoid bodies. We characterized the 25-day procedure by morphologically examining the differentiating lentoid bodies (Fig. 1). On day 6, we isolated epithelial-like lens-fated cells present at the periphery of both hESC and iPSC colonies and transferred them to new Matrigel (Corning) coated 35 mm plates (Fig. 1). A fried egg like morphology started to appear as early as on day 8 for differentiating hESCs and PBMC-originated, iPSCs. In contrast to differentiating hESCs, PBMC-originated, iPSCs exhibited decreased fried egg-like structures. On day 10, differentiating hESCs and PBMC-originated, iPSCs unable to form fried egg-like appearance were mechanically discarded. On day 15, lens epithelial-like cells were started to differentiate into fiber cell-like cells in the middle of differentiating lentoid bodies (Fig. 1). Finally, on day 25, transparent lentoid bodies with lens-like morphological appearance were observed (Fig. 1).

### Gene expression analysis by qRT-PCR

**Figure 2:**
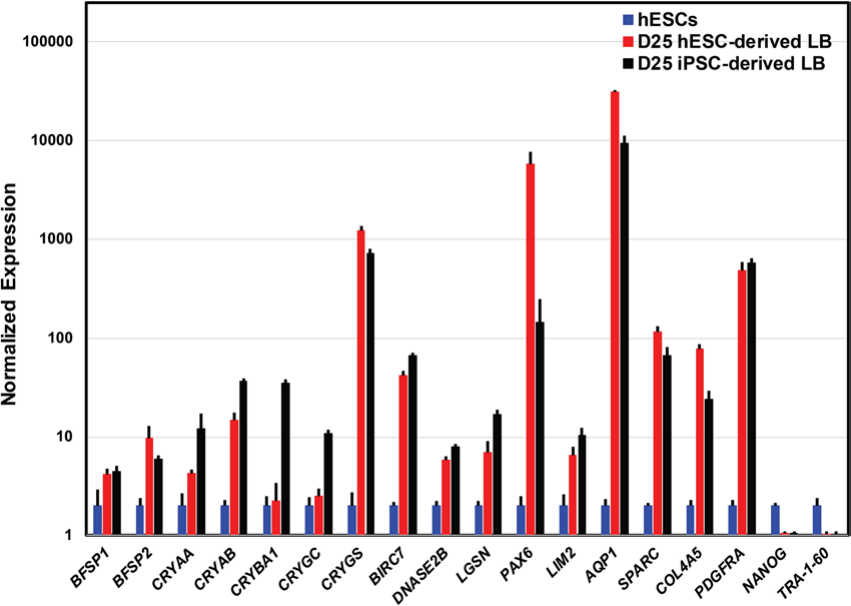
Gene expression analysis of lens- and pluripotency-associated markers in H9 human embryonic stem cell (hESC)- and peripheral blood mononuclear cell (PBMC)-originated, induced pluripotent stem cell (iPSC)-derived lentoid bodies (LB). The expression level of 16 lens-associated markers (*BFSP1, BFSP2*, *CRYAA*, *CRYAB*, *CRYBA1*, *CRYGC, CRYGS*, *BIRC7*, *DNASE2B*, *LGSN*, *PAX6, LIM2*, *AQP1*, *SPARC*, *COL4A5*, and *PDGFRA*) and two pluripotent markers i.e*. NANOG* and *TRA-1-60* were analyzed by quantitative real-time PCR (qRT-PCR) in hESC- and PBMC-originated, iPSC-derived LB. Error bars represent the standard deviation of two independent experiments and each experiment was performed with three biological replicates. Note: Expression of all markers is normalized against *GAPDH* and all values are relative to the respective expression of hESCs.

The qRT-PCR based gene expression analysis of lens- and pluripotency-associated markers was performed to investigate lens-specific characteristics in hESC- and iPSC-derived lentoid bodies at differentiation day 25 (Fig. 2). On day 25, the expression of pluripotent markers (*NANOG* and *TRA-1-60*) was significantly down-regulated compared with their respective expression in hESCs, confirming the loss of pluripotency (Fig. 2). In parallel, we identified increased expression levels of lens-associated markers strongly supporting the differentiation of hESCs and iPSCs into lens-specific cells (Fig. 2). The iPSC-derived lentoid bodies revealed an increased expression of lens-associated markers (*CRYAA*, *CRYAB*, *CRYBA1*, *CRYGC*, and *LGSN* compared to hESC-derived lentoid bodies (Fig. 2). Whereas, a decreased expression levels of *BFSP2*, *PAX6*, *AQP1*, and *COL4A5* were observed in iPSC-derived lentoid bodies compared to hESC-derived lentoid bodies (Fig. 2). Both hESC- and iPSC-derived lentoid bodies exhibited similar expression levels of *BFSP1, CRYGS*, *BIRC7*, *DNASE2B*, *LIM2*, *SPARC*, and *PDGFRA* (Fig. 2). The high expression of lens epithelial cell-associated markers (*AQP1*, *SPARC*, *COL4A5*, and *PDGFRA*) compared to lower expression levels of lens fiber cell-associated markers (*BIRC7*, *DNASE2B*, and *LGSN*) in hESC- and iPSC-derived lentoid bodies suggest that lentoid bodies at day 25 probably mimics an early differentiation stage of ocular lens development.

### Transmission electron microscopy

**Figure 3:**
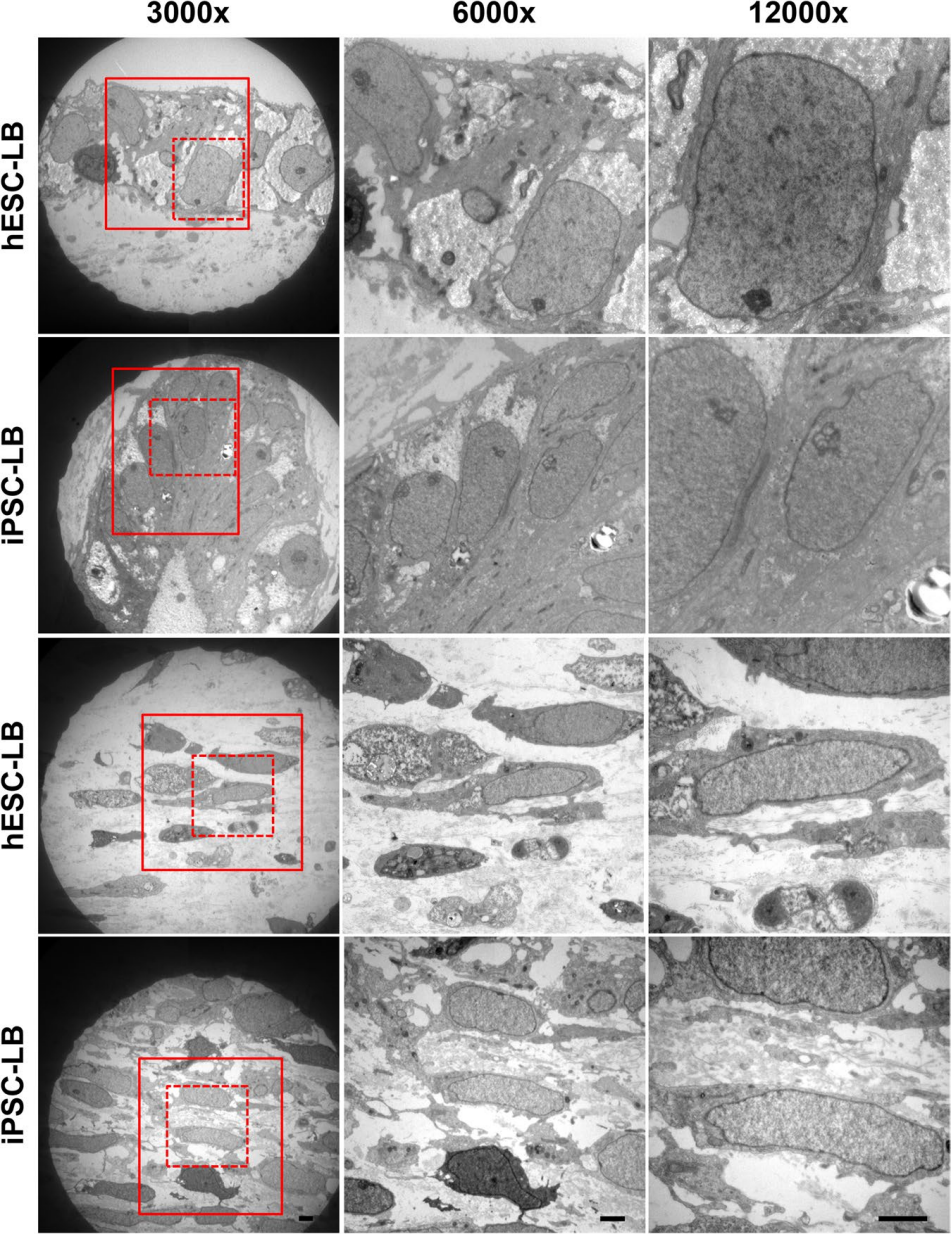
Transmission electron micrographs of H9 human embryonic stem cell (hESC)- and peripheral blood mononuclear cell (PBMC)-originated, induced pluripotent stem cell (iPSC)-derived lentoid bodies. Ultrastructure analysis revealed closely packed lens epithelial-like and fiber-like cells in lentoid bodies. Note: The images are of 3000x, 6000x and 12000x magnifications and scale bars represent 2 μm.

The hESC- and iPSC-derived lentoid body transmission electron microscopy showed a compact arrangement of lens epithelial-like cells with rectangle morphology having regular nuclei and organelles (Fig. 3). In addition, differentiating fiber-like cells with degenerating cytoplasmic profiles were observed adjacent to the lens epithelial-like cells in both hESC- and iPSC-derived lentoid bodies (Fig. 3).

### Next generation RNA sequencing (RNA-Seq)

To investigate the transcriptional landscape of hESC- and iPSC-derived lentoid bodies, next generation RNA-Seq was performed. The RNA-Seq analyses identified the expression (≥0.659 RPKM) of 13,975 and 14,003 genes in hESC- and iPSC-derived lentoid bodies, respectively (Supplementary Tables 1 and 2), which suggests that ~70% of the total human protein-coding transcriptome is expressed in lentoid bodies. All raw and processed sequencing data reported in this manuscript have been deposited in NCBI’s Gene Expression Omnibus^7^, and are accessible through GEO series accession number GSE111071.

**Figure 4:**
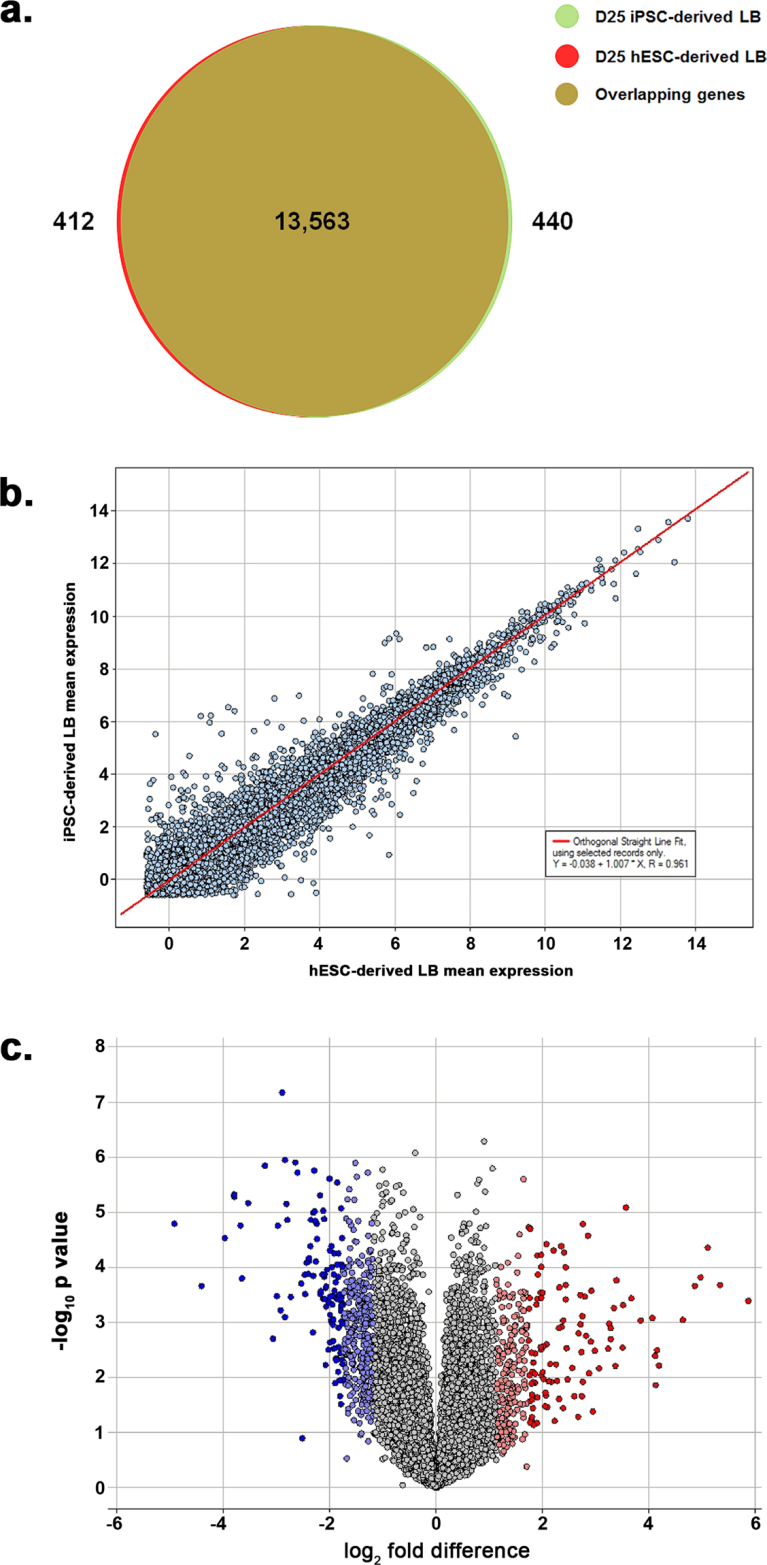
Characterization of H9 human embryonic stem cell (hESC)- and peripheral blood mononuclear cell (PBMC)-originated, induced pluripotent stem cell (iPSC)-derived lentoid body (LB) transcriptome datasets. (**a**) Venn diagram illustrating the overlap between the transcriptomes of hESC- and iPSC-derived LB. A total of 13,975 and 14,003 genes were identified in hESC- and iPSC-derived LB, respectively. The red and green represent genes identified in hESC- and iPSC-derived LB, respectively. Whereas, the overlap between red and green represents 13,563 genes common in both hESC and iPSC-derived LB transcriptomes. (**b**) A scatter plot showing normalized expression values of both hESC- and iPSC-derived LB datasets, which illustrates a high correlation among both datasets (Pearson correlation R = 0.961). The RPKM (reads per kilobase per million mapped reads) normalization method was used to measure the gene expression in both datasets and was transformed to a logarithmic scale (log_2_). Note: Each circle represents a gene. (**c**) Volcano plot representation of differentially expressed genes identified in iPSC-derived LB compared with hESC-derived LB. The analysis revealed 707 differentially expressed genes (>2 Std. Dev.) including 416 down- and 291 up-regulated genes in iPSC-derived LB, compared to hESC-derived LB. The fold changes are represented in a log_2_ scale depicted on the x-axis, whereas the −log_10_ p-value is depicted on the y-axis (the use of −log values mean that transcripts with greater statistical significance are higher in the plot). Genes that are significantly up-regulated are highlighted in red and light red, and those with significant down-regulated are highlighted in blue and light blue. **Note:** Std. Dev.: standard deviation.

We performed a comparative analysis to identify overlapping genes in hESC- and iPSC-derived lentoid body transcriptomes. The analysis revealed 13,563 (>96%) genes shared in both transcriptomes (Fig. 4a, Supplementary Tables 1 and 2). Furthermore, a high correlation (Pearson coefficient = 0.961) was identified among hESC- and iPSC-derived lentoid body transcriptomes (Fig. 4b). Next, we investigated the differentially expressed genes present in both transcriptomes. Among the genes common in both transcriptomes, 12,856 (~95%) exhibited a quantitatively similar expression profile (≤2 Standard deviations (Std. Dev.); Supplementary Table 3). We only identified a small fraction (~5%) of the transcriptome that exhibited a differential expression (>2 Std. Dev.) in the two datasets. We identified 707 genes including 416 down- and 291 up-regulated in iPSC-derived lentoid bodies, compared to hESC-derived lentoid bodies (Fig. 4c & Supplementary Table 3).

**Figure 5:**
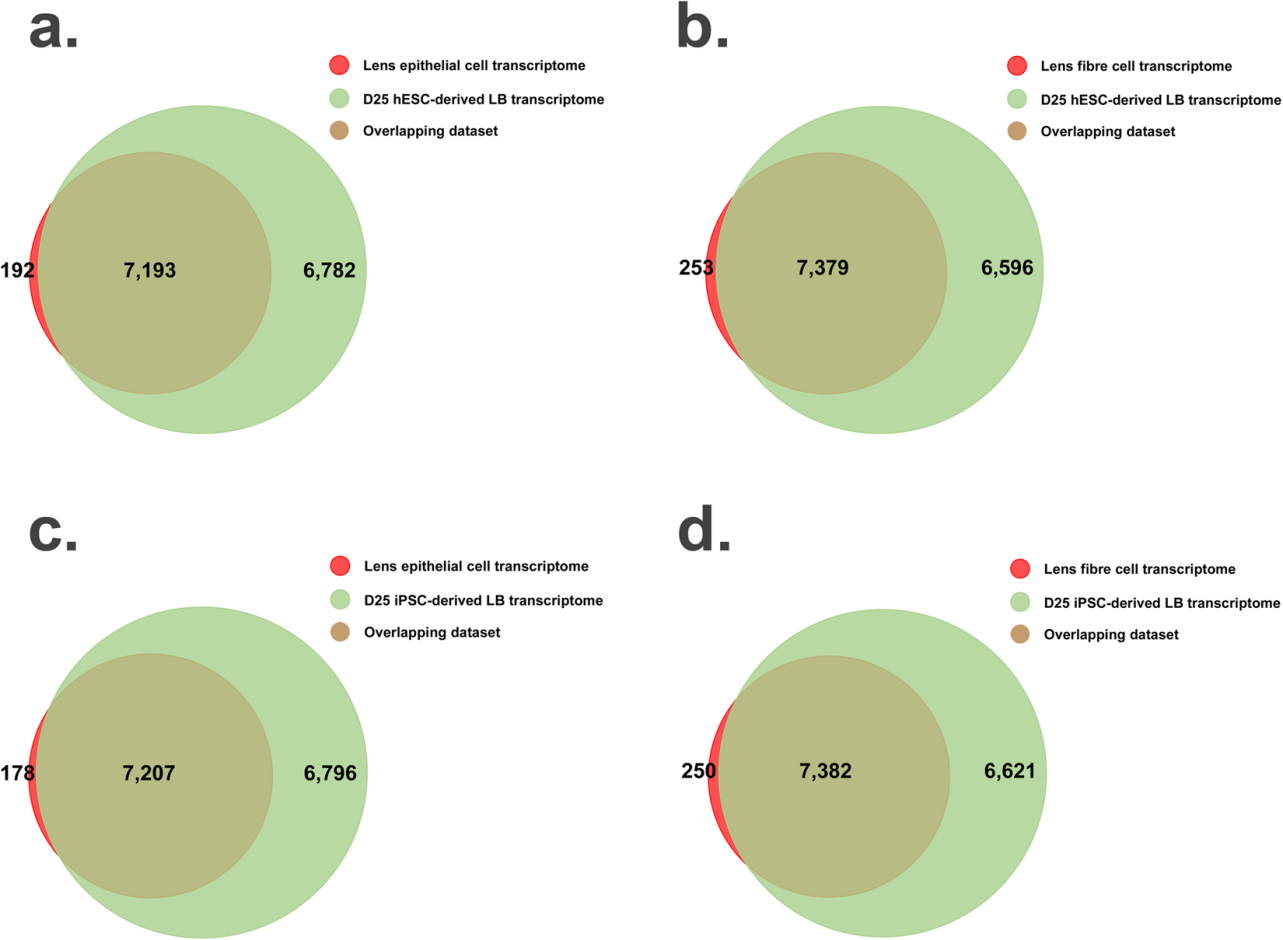
Comparative transcriptome analyses of H9 human embryonic stem cell (hESC)- and peripheral blood mononuclear cell (PBMC)-originated, induced pluripotent stem cell (iPSC)-derived lentoid bodies at day 25 (D25) with mouse lens epithelial and fiber cell transcriptome datasets. (**a**) The Venn diagram illustrates an overlap between hESC-derived lentoid body and mouse lens epithelial cell transcriptome datasets. The green and red represent genes identified in iPSC-derived lentoid bodies and mouse lens epithelial cell transcriptomes, respectively, whereas, the overlap represents genes common in both datasets. (**b**) The Venn diagram illustrates an overlap between hESC-derived lentoid body and lens fiber cell transcriptome datasets. The green and red represent genes identified in hESC-derived lentoid body and lens fiber cell transcriptomes, respectively, whereas, the overlap represents genes common in both datasets. (**c**) The Venn diagram illustrates an overlap between iPSC-derived lentoid body and mouse lens epithelial cell transcriptome datasets. The green and red represent genes identified in iPSC-derived lentoid body and lens epithelial cell transcriptomes, respectively, whereas, the overlap represents genes common in both datasets. (**d**) The Venn diagram illustrates an overlap between iPSC-derived lentoid body and lens fiber cell transcriptome datasets. The green and red represent genes identified in iPSC-derived lentoid body and mouse lens fiber cell transcriptomes, respectively, whereas, the overlap represents genes common in both datasets.

Next, a comparative analysis of hESC- and iPSC-derived lentoid body transcriptomes was performed with published mouse lens epithelial and fiber cell transcriptomes. Hoang and colleagues reported the transcriptome profile of lens epithelial and fiber cells isolated from newborn mice^8^. The comparative analyses illustrated >97% of mouse epithelial cell transcriptome overlaps with hESC-and iPSC-derived lentoid body transcriptome datasets (Fig. 5a,c). Likewise, the analyses revealed >96% of mouse fiber cell transcriptome present in hESC- and iPSC-derived lentoid body transcriptome datasets (Fig. 5b,d).

## Discussion

Here, we report comprehensive transcriptome profiles of hESC- and iPSC-derived lentoid bodies using next-generation RNA-Seq. Our datasets confirm comparable expression profiles of both hESC- and iPSC-derived lentoid body transcriptomes at day 25, strongly suggesting that both transcriptomes are largely equivalent. In addition, the use of patient-specific iPSC-derived lentoid bodies may provide a handful tool for understanding the process of cataractogenesis and possible therapeutic intervention. These datasets strongly advocate for the use of pluripotent stem cell-derived lentoid bodies for understanding the mechanism of lens morphogenesis. To the best of our knowledge, this is the first report on the comparative transcriptome analysis of hESC- and iPSC-derived lentoid bodies to understand the molecular landscape of pluripotent stem cells derived lentoid bodies.

The lentoid body transcriptome datasets revealed a diverse catalog of genes, including crystallin, osmolarity related, heat shock, and cytoskeleton-associated genes (Supplementary Tables 1 and 2). It has been reported that *PAX6* is critical for lens induction and express during lens development^9–11^. Both hESC- and iPSC-derived lentoid body transcriptome datasets revealed *PAX6* expression (Supplementary Tables 1 and 2). In addition, the qRT-PCR analysis also confirmed the comparable expression of *PAX6* in hESC- and iPSC-derived lentoid bodies (Fig. 2). We identified 11 different crystallin genes, including *CRYAB*, *CRYBB1*, *CRYBB2*, *CRYBB3*, *CRYBG2, CRYBG3,CRYGS*, *CRYL1*, *CRYM*, *CRYZ*, and *CRYZL1* (Supplementary Tables 1 and 2). The low expression levels of crystallin in both hESC- and iPSC-derived lentoid bodies indicate that lentoid bodies at day 25 probably mimic an early ocular lens development time point. This notion is further validated by high expression of lens epithelial cells-associated markers (*AQP1*, *SPARC*, *COL4A5*, and *PDGFRA*) compared to lower expression of lens fiber cell-associated markers (*BIRC7*, *DNASE2β*, and *LGSN*) in lentoid bodies. The ultrastructure examination of hESC- and iPSC-derived lentoid bodies revealed both lens epithelial and lens fiber-like cells. Similarly, Fu and colleagues reported tightly packed lens epithelial and fiber-like cells in human urinary epithelial cell-originated, iPSC-derived lentoid bodies^4^.

The ocular lens development is a complex process originating from a single layer of epithelial cells that proliferate and differentiate into lens fiber cells^12^. The differentiating lens fiber cells possess a unique expression profile characterized by an abundance of crystallin, lens cytoskeleton, and heat shock proteins^8,13^. Low expression of *CRYBB2* was identified in both hESC- and iPSC-derived lentoid bodies whereas expression of *CRYBB1* and *CRYBB3* was only identified in hESC-derived lentoid bodies (Supplementary Tables 1 and 2). The high expression of *CRYBB2* was observed in isolated mouse fiber cells and postnatal mouse lens^8,13,14^.

Secreted frizzled-related proteins (Sfrp) play an important role in lens development with strong expression in lens fiber cells^15^. We identified the expression of *SFRP1* and *SFRP2* in the lentoid body transcriptomes (Supplementary Tables 1 and 2). In addition, the expression of lens fiber cell-associated markers (*PGAM2*, *LSAMP*, *CAPRIN2*, *TDRD7*, *LCTL*, and *BIRC7*) were identified in hESC- and iPSC-derived lentoid bodies (Supplementary Tables 1 and 2). Heat shock proteins are among the most important structural proteins in the eye lens^16^. The hESC- and iPSC-derived lentoid body transcriptome revealed 70 plus different heat shock genes, including high expression of *HSPB1*, *HSP90AA1*, *HSP90AB1*, *DNAJB1*, *HSF1* and *HSF4* (Supplementary Tables 1 and 2).

We previously published a comprehensive transcriptome profile of developing mouse lens at different time points^13^. Hoang and colleagues reported the transcriptome profile of lens epithelial and fiber cells isolated from newborn mice^8^. The comparative analyses of mouse lens epithelial and fiber cell transcriptomes with lentoid body transcriptome datasets illustrated the majority of mouse epithelial and fiber cell genes overlap with hESC- and iPSC-derived lentoid body transcriptome datasets.

In conclusion, we report comprehensive transcriptome profiles of hESC- and iPSC-derived lentoid bodies exhibiting comparable expression profiles. These datasets strengthen the notion that pluripotent stem cell-derived lentoid bodies may be useful for understanding the mechanism of lens morphogenesis and age-related cataracts. In addition, patient-specific iPSC-derived lentoid bodies are useful to investigate the process of cataractogenesis and examining the efficacy of anti-cataract drug screening.

## Materials and Methods

### Subjects and clinical ascertainment

Institutional Review Board (IRB) approval for research involving human subjects was obtained from the Office of Human Subjects Research, Johns Hopkins University School of Medicine. Likewise, IRB approval for research involving pluripotent stem cells was obtained from the Institutional Stem Cell Research Oversight Committee (ISCRO), Johns Hopkins University School of Medicine. The participating subject gave informed consent consistent with the tenets of the Declaration of Helsinki.

### Isolation of PBMCs and generation of iPSCs

The PBMCs were isolated according to the procedure published by Agu and colleagues^17^. Briefly, 10 ml of human peripheral blood was taken in a 50 ml conical tube containing 0.5 M EDTA and diluted with 2 volumes of phosphate-buffered saline (PBS). The mixture was gently layered on 15 ml of histopaque (MilliporeSigma, Burlington, MA, USA) and centrifuged at 400 g for 30 minutes (without brake).

The mononuclear cells layer was gently collected in 50 ml tube, washed twice with PBS by centrifugation at 300 g for 10 minutes, and 1 × 10^6^ PBMCs were cryopreserved in a 1 ml cryopreservation medium comprising of 10% dimethyl sulphoxide (DMSO; MilliporeSigma) and 90% fetal bovine serum (MilliporeSigma).

The cryopreserved PBMCs were reprogramed using a Sendai virus delivery system, according to the manufacturer’s instructions (Cytotune 2.0; Life Technologies, Carlsbad, CA, USA). Briefly, the PBMC vial was removed from liquid nitrogen and thawed at 37 °C in a water bath. Subsequently, the PBMCs were washed with medium (StemSpan; STEMCELL Technologies, Inc.) and cultured in StemSpan medium supplemented with 100 ng/ml Feline McDonough Sarcoma-like tyrosine kinase 3 ligand (FLT-3L), 100 ng/ml stem cell factor (SCF), 20 ng/ml interleukin-3 (IL-3), and 20 ng/ml interleukin-6 (IL-6), termed complete medium hereafter, in a humidified incubator at 37 °C supplemented with 5% CO_2_ for 4 days. Approximately, 5 × 10^5^ cells/ml PBMCs were collected in a round-bottom tube in 1 ml complete medium, followed by infection by reprogramming viral particles at a multiplicity of infection (MOI) of 5, 5, and 3 (KOS MOI = 5; hc-MYC MOI = 5; hKLF4 MOI = 3). The infected cells were centrifuged at 1000 g for 30 minutes at room temperature. The cells were resuspended in 1 ml complete medium, transferred to a single well of a 12-well plate, and cultured for 3 days in complete medium. Subsequently, the cells were transferred to mouse embryonic fibroblast-coated plates for 3 days with complete medium and finally transferred to Dulbecco’s modified Eagle’s medium-F12 (DMEM-F12) supplemented with 20% knockout serum replacement (KSR; Life Technologies) and 20 ng/ml basic fibroblast growth factor (bFGF; Life Technologies) until an embryonic stem cell-like colony was formed. An embryonic stem cell-like putative iPSC colonies were selected and cultured on a Matrigel-coated (Corning, Bedford, MA, USA) plate in mTeSR1 medium (STEMCELL Technologies, Inc., Vancouver, BC, Canada) for characterization.

### Culturing of hESCs

The H9 hESCs (WiCell Research Institute, Madison, WI, USA) were cultured in mTeSR1 medium (StemCell Technologies Inc., Canada) in feeder-free conditions on a Matrigel (Corning, Bedford, MA, USA) coated 35 mm plates. The cells were passaged using 0.5 mM EDTA in PBS every 5–6 days and the culture medium was changed daily.

### Phase-contrast microscopy

Phase-contrast microscopy was performed using a Zeiss Axio Observer A1 inverted microscope (Zeiss, Germany) equipped with Q-Capture software (QImaging, Surrey, Canada).

### Characterization of PBMC-originated, iPSCs

The PBMC-originated, iPSCs were characterized by phase-contrast microscopy, flow cytometry, and qRT-PCR. Approximately 2 × 10^5^ cells were labeled for the flow cytometery with TRA-1-60 and SSEA4 (Cell signaling technology, Danvers, MA) primary antibodies for 1 hour at 4 °C followed by treatment with FITC-conjugated goat anti-mouse IgG antibody (against TRA-1-60 and SSEA4; Millipore, Billerica, MA) for 40 min at 4 °C. 10,000 events were acquired for each sample using a guava easyCyte flow cytometer (Millipore BD Science, Billerica MA).

For qRT-PCR analysis, RNA from the iPSCs was extracted using TRIzol reagent (Invitrogen; Carlsbad, CA) and the first-strand cDNA was synthesized using the Superscript III kit (Invitrogen Carlsbad, CA) according to the manufacturer’s instructions. qRT-PCR was performed on the STEP ONE ABI Real-Time PCR System. The expression of pluripotent markers (*NANOG*, *OCT4*, *SOX2*, and *TRA-1-60*), were quantitated using Power SYBR Green PCR Master Mix (Life Technologies). Glyceraldehyde-3-phosphate dehydrogenase (*GAPDH*) was used as an endogenous control. The delta–delta CT method was used to determine the relative expression, normalized against *GAPDH*^18,19^. All the primers used in the qRT-PCR analysis were designed using Real-time PCR tool (Integrated DNA Technologies) and will be provided upon request.

### Differentiation of hESCs and iPSCs into lentoid bodies

The lentoid bodies were generated from H9 hESCs and PBMC-originated, iPSCs using the “fried egg” method according to the procedure reported by Fu and colleagues with brief modifications^4^. Briefly, 60–70 hESC and iPSC colonies with ~25 cells in each colony, were cultured in mTeSR1 medium (Stemcell Technologies) on a Matrigel (Corning) coated 35 mm plate in a humidified incubator at 37 °C supplemented with 5% CO_2_. The pluripotent stem cells were treated with mTeSR1 medium containing 100 ng/ml of mouse recombinant Noggin (R&D Systems, Minneapolis, MN) four hours post-plating, which continued for six days (day 0 to day 6).

On day 6, cell colonies comprising of ~60–65 differentiating pluripotent stem cells along with adjacent epithelial-like cells were mechanically isolated and reseeded in Matrigel (Corning) coated 35 mm plates containing a cocktail of 100 ng/ml of bFGF (R&D Systems), 20 ng/ml bone morphogenetic protein 7 (BMP7; R&D Systems) and 20 ng/ml bone morphogenetic protein 4 (BMP4; R&D Systems) for nine days (day 6 to day 15). Importantly, on day 10, cell clusters that did not form “fried egg” morphologies were mechanically discarded. On day 15, cell clusters were treated with 100 ng/ml bFGF along with 20 ng/ml Wnt3a (PeproTech, Rocky Hill, NJ, USA) until the day of harvesting. The differentiating lentoid bodies were harvested on day 25 for subsequent analyses including transcriptome profiling.

### qRT-PCR analysis

The H9 hESC- and iPSC-derived lentoid bodies at day 25 were preserved in three distinct pools (each containing five lentoid bodies) serve as three biological replicates. The total RNA from the hESC- and iPSC-derived lentoid bodies at day 25 was extracted using TRIzol reagent (Invitrogen; Carlsbad, CA) as mentioned earlier. The preparation of first-strand cDNA and qRT-PCR analysis was performed using the same procedure as mentioned above. The expression levels of 16 lens-associated markers (*BFSP1, BFSP2*, *CRYAA*, *CRYAB*, *CRYBA1*, *CRYGC, CRYGS*, *BIRC7*, *DNASE2B*, *LGSN*, *PAX6, LIM2*, *AQP1*, *SPARC*, *COL4A5*, and *PDGFRA*) and two pluripotent markers i.e*. NANOG* and *TRA-1-60* were analyzed by qRT-PCR in hESC- and iPSC-derived lentoid bodies. *GAPDH* was used as an endogenous control. All the primers used in the qRT-PCR analysis were designed using Real-time PCR tool (Integrated DNA Technologies) and are available upon request.

### Transmission electron microscopy

The hESC- and iPSC-derived lentoid bodies at differentiation day 25 were quickly rinsed with 1X PBS for 30 seconds, and PBS was replaced with 2.5 ml of 2.5% glutaraldehyde in 0.1M phosphate (Sorenson’s) and 5 mM MgCl_2_ buffer (pH 7.4) and culture dishes were allowed to slowly rock for 4 hours at room temperature. Subsequently, lentoid bodies were post-fixed with 1% OsO_4_ in phosphate buffer (0.1 M, pH 7.0) for 2 hours with slow rocking at 4 °C. Samples were then rinsed in cold 100 mM maleate buffer containing 3% sucrose (336 mOsmols) for 30 minutes and en-bloc stained with filtered 2% uranyl acetate in maleate containing sucrose, in the dark at 4 °C. Culture dishes were then dehydrated at 4 °C up to 70% ethanol, transferred to room temperature and further dehydrated to 100% ethanol. Plates were then infiltrated with pure resin (Eponate 12, Ted Pella) and cured for three days at 37 °C, and one day at 60 °C. Hardened blocks were removed from the plastic plates and discs were punched out and mounted to blank upon blocks. Blocks were then trimmed and sectioned. Sections were cut on a Reichert Ultracut E microtome with a Diatome Diamond knife (45 degrees). 80 nm sections were picked up on formvar-coated 1 × 2 mm copper slot grids and stained with methanolic uranyl acetate followed by lead citrate. Grids were viewed on a Hitachi H 7600 TEM operating at 80 kV and digital images captured with an XR50- 5-megapixel CCD by AMT.

### RNA-Seq library preparation and next-generation sequencing

Next-generation RNA-Seq of H9 hESC- and iPSC-derived lentoid bodies was performed as described previously^20^. Briefly, four biological replicates (each replicate containing two lentoid bodies) for hESC- and iPSC-derived lentoid bodies at day 25 were used for RNA-Seq library preparation. Total RNA was isolated from each sample using the Quick-RNA™ MicroPrep kit (Zymo Research; Irvine, CA). The extracted RNA was examined using a NanoDrop Lite spectrophotometer (Thermo Fisher Scientific) and RNA 6000 Pico kit on an Agilent 2100 Bioanalyzer (Agilent; Palo Alto, CA), respectively.

Total RNA was subjected to RNA-Seq library preparation using NEBNext Ultra II RNA Library Prep Kit for Illumina (NEB, Ipswich, MA). Approximately, 1.0 µg of high-quality total RNA (≥8.0 RIN Score) was used for polyadenylated RNA selection using oligo dT beads, followed by the fragmentation of selected polyadenylated RNA. The fragmented RNA was used as a template for cDNA synthesis by reverse transcriptase with random primers. The cDNA was further converted into double-stranded DNA that was end-repaired to incorporate the specific index adapters for multiplexing, followed by a purification step and amplification for 15 cycles. The amplified libraries were examined on an Agilent 2100 Bioanalyzer (Agilent) and quantitative PCR (qPCR) was completed according to the manufacturer’s instructions.

RNA-Seq libraries with unique index sequences were pooled in an equimolar ratio, and a final size selection of 400–500 bp was performed. After the quality control confirmation, eight RNA-Seq bar-coded pooled libraries were sequenced (2 × 100 bp) in a single lane on a HiSeq 2500 (rapid run mode) genome analyzer. The base calls were assigned through Illumina Real-Time Analysis software (Ver. 1.17.20) and binary base call (BCL) files were converted to a flat-file format (qseq.txt) using Illumina BCL Converter software (Ver. 1.9.4). Qseq.txt files were de-multiplexed to single sample FASTQ files using de-multiplexer software written at the Center for Inherited Disease Research (CIDR), Johns Hopkins University as part of CIDRSeqSuite (Ver. 7.1.0; unpublished).

### Bioinformatics and data analysis

The raw RNA-Seq reads (FASTQ) were processed and analyzed using Lasergene Genomics Suite (DNASTAR, Madison, WI, USA). The paired-end reads were assembled with SeqMan NGen (Ver. 12), using default parameters and aligned to the human reference genome (GRCh38.p11). The ArrayStar (Ver. 12; DNASTAR) was used for normalization, differential gene expression and statistical analysis of mapped paired-end reads using the default parameters. The expression data quantification and normalization were calculated using the RPKM value for each gene^21^.

In parallel, we examined differential expression in hESC- and iPSC-derived lentoid bodies using Spotfire DecisionSite with Functional Genomics (TIBCO Spotfire, Boston, MA). Expression differential between hESC- and iPSC-derived lentoid bodies were analyzed to determine standard deviation from their mean of 0, which represents no change. We chose > 2 Std. Dev. the cutoff value for differentially expressed genes.

## Supplementary information

Supplementary Info

Supplementary Table 1

Supplementary Table 2

Supplementary Table 3
