# Supplementary material for: Comparative transcriptome analysis of hESC- and iPSC-derived lentoid bodies
Source: Sci Rep. 2019 Dec 6;9:18552. doi: 10.1038/s41598-019-54258-z (PMC6898283; doi:10.1038/s41598-019-54258-z)
Supplement: Supplementary file 1 — Supplementary Info [file 41598_2019_54258_MOESM1_ESM.pdf]

Comparative transcriptome analysis of hESC- and iPSC-derived lentoid bodies

Muhammad Ali,[1] Firoz Kabir,[1] Jason J. Thomson,[2] Yinghong Ma,[2] Caihong Qiu,[2] Michael Delannoy,[3] Shahid Y. Khan,[1] S. Amer Riazuddin [1]

[1]The Wilmer Eye Institute, Johns Hopkins University School of Medicine, Baltimore MD 21287; [2]Yale Stem Cell Center, Yale University School of Medicine, New Haven CT 06520; [3]Department of Cell Biology and Imaging Facility, Johns Hopkins University School of Medicine, Baltimore MD

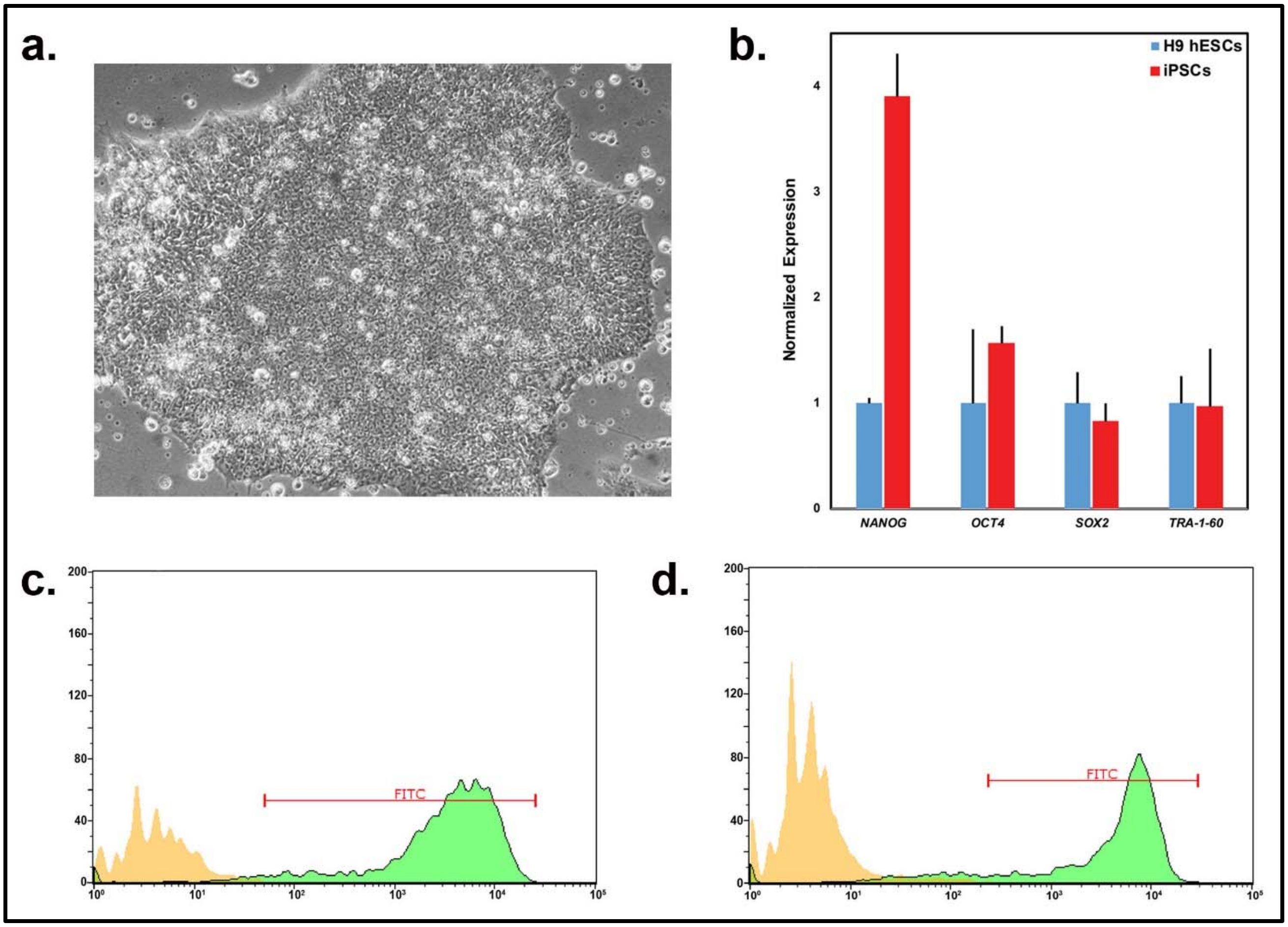

Supplementary Figure 1: Characterization of peripheral blood mononuclear cell (PBMC)-originated, induced pluripotent stem cells (iPSCs). **a.** Phase-contrast image of PBMC-originated, iPSCs (magnification 10×). **b.** Gene expression analysis of pluripotent-associated markers i.e. *NANOG, OCT4, SOX2* and *TRA-1-60* by qRT-PCR. **c-d.** Flow cytometric analysis of PBMC-originated, iPSCs for SSEA4 and TRA-1-60. The yellow and green areas represent the control isotype and expression of iPSCs-associated markers, respectively.

Supplementary Table 1: A complete list of genes identified in H9 human embryonic stem cell (hESC)-derived lentoid bodies on day 25 of differentiation. The gene expression data were normalized by calculating the RPKM (reads per kilobase per million mapped reads; RRKM= calculated as total exon reads/mapped reads in millions × exon length in kb) for each gene. A cut-off value of ≥ 0.659 RPKM was established as an expression threshold for each gene.

Supplementary Table 2: A complete list of genes identified in peripheral blood mononuclear cell (PBMC)-originated, induced pluripotent stem cell (iPSC)-derived lentoid bodies on day 25 of differentiation. The gene expression data were normalized by calculating the RPKM (reads per kilobase per million mapped reads; RRKM= calculated as total exon reads/mapped reads in millions × exon length in kb) for each gene. A cut-off value of ≥ 0.659 RPKM was established as an expression threshold for each gene.

Supplementary Table 3: A complete list of differentially expressed genes identified in induced pluripotent stem cell (iPSC)-derived lentoid bodies compared with H9 human embryonic stem cell (hESC)- derived lentoid bodies on day 25 differentiation.
